# Venous Thromboembolism in Prader–Willi Syndrome: A Questionnaire Survey

**DOI:** 10.3390/genes10070550

**Published:** 2019-07-19

**Authors:** Ann M. Manzardo, Janalee Heinemann, Barbara McManus, Carolyn Loker, James Loker, Merlin G. Butler

**Affiliations:** 1 Departments of Psychiatry & Behavioral Sciences and Pediatrics, University of Kansas Medical Center, Kansas City, KS 66160, USA; 2 Prader-Willi Syndrome Association (USA), Sarasota, FL 34238, USA; 3 Department of Pediatrics, Bronson Hospital, Western Michigan University, Kalamazoo, MI 49008, USA

**Keywords:** Prader–Willi syndrome, thromboembolism, risk factors, vasculitis, blood clots

## Abstract

Prader–Willi Syndrome Association (USA) monitors the ongoing health and welfare of individuals with Prader–Willi syndrome (PWS) through active communication with members by membership surveys and data registries. Thromboembolism and blood clots have emerged in clinical studies as significant risk factors for injury and death in PWS. A 66-item questionnaire was developed by a panel of PWS medical and scientific experts, with input from Prader–Willi Syndrome Association (USA) leadership, so as to probe their membership on the frequency, risk, and protective factors for venous thromboembolism, pulmonary embolism, and related findings. The characteristics of those with and without a reported history of blood clots and related health factors were tabulated and analyzed. Responses were obtained for 1067 individuals with PWS (554 females and 513 males), and 38 (23 females and 15 males) had a history of blood clots. The individuals with clots did not differ by gender, but were significantly older 32.8 ± 15 years vs 20.4 ± 13 years, and were more likely to have a reported history of obesity (76%), edema (59%), hypertension (24%), vasculitis (33%), and family history of blood clots (33%) than those without clots. Growth hormone treatment was more common in individuals without clots. The risk factors for thromboembolism in PWS overlap those commonly observed for the general population.

## 1. Introduction

Prader–Willi syndrome (PWS) is a complex neurodevelopmental genetic disorder as a result of errors in genomic imprinting. Characteristics include infantile hypotonia; hypogonadism and hypogenitalism; poor suck reflex with feeding difficulties during infancy; developmental delay with mental deficiency; growth and other hormone deficiencies with small hands and feet; short stature; a particular facial pattern and behavioral problems including skin picking, obsessive compulsions, and hyperphagia leading to obesity, if not externally controlled [1,2,3,4]. PWS is considered the most common genetic cause of marked obesity in humans, with an estimated 350,000 to 400,000 people worldwide, and more than 15,000 individuals in the USA [5]. The incidence is between 1 in 10,000–20,000. A paternally derived chromosome 15q11-q13 deletion is seen in about 60% of individuals, and maternal disomy 15 (both chromosome 15s inherited from the mother) is seen in about 35% of cases, while the remaining individuals have a defect of the chromosome 15 imprinting center controlling the imprinted genes or other chromosome 15 abnormalities (translocations or inversions) [6].

Recent reviews in causes of death and survival trends in Prader–Willi syndrome have increased our awareness of the mortality and risk factors contributing to death, specifically blood clots and pulmonary embolism [7,8]. Pulmonary embolism (PE) is the fourth most common reported cause of death in PWS, reported in over 400 deceased patients studied from the PWSA (USA) registry database, and blood clots as a cause of death have also been reported in clinical trials [7,9]. PE is also a recognized cause of death in obese individuals, as well as in our society. Beckman et al. [10,11]. further estimated that more than 300,000 Americans have had an episode of deep venous thrombosis (DVT) or pulmonary embolism, and it is now recognized as a growing public health concern associated with considerable morbidity and mortality. It affects all races, ethnicities, age groups, and both genders. Other known risk factors include an advanced age, surgery, and immobility. Learning more about the burden and causes of venous thromboembolism (VTE), and better surveillance by the public and medical community with advanced research, has the potential to prevent or at least reduce morbidity and death. This knowledge could also apply to obesity-related genetic disorders such as PWS, possibly at a young age. For example, a newborn female with PWS has been reported in the literature, with neonatal cerebral venous thrombosis [12]. 

Previous studies have shown that choking, swallowing difficulties, excessive eating, and obesity, if not externally controlled, can occur in early childhood, and gastric necrosis in adults, leading to death in PWS [2,13]. Furthermore, factors reported recently that contribute to mortality in PWS include increased weight, heart problems, sleep apnea and other respiratory complications, diabetes, osteoporosis, high pain tolerance, severe skin picking, and the duration of growth hormone use [8,14]. Hence, obesity and its subsequent consequences are primary contributors to mortality in PWS. 

We developed a large nationwide survey to entertain questions about the natural history and factors related to thromboembolism and blood clots as a cause of death in PWS. 

## 2. Materials and Methods

**Figure 1:**
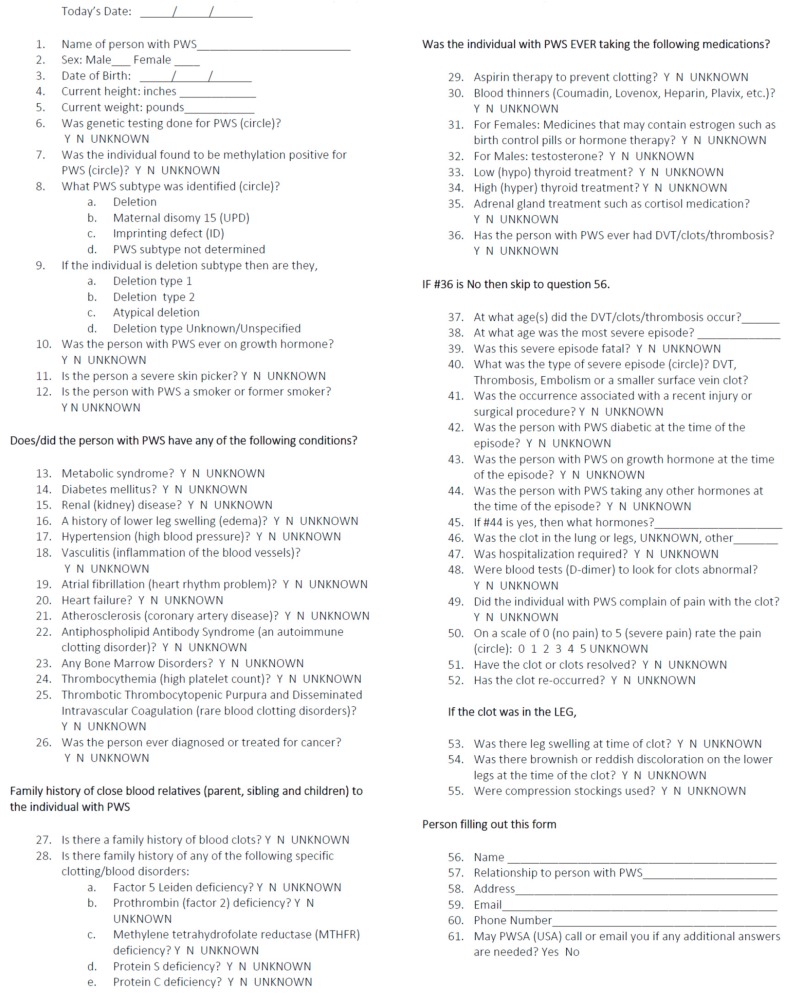
Vascular Blood Clots, Deep Vain Thrombosis and/or Thrombosis in Prader-Willi Syndrome Questionnaire.

A health-related questionnaire was developed so as to assess the occurrence of vascular issues, blood clots, deep vein thrombosis, and/or pulmonary embolism in PWS, and to characterize the contributing factors based on the frequency of known risk factors from PWS and the general population. The survey material was compiled by a team of researchers and health care providers experienced in PWS in consultation, along with PWSA (USA) leadership. The resulting 66-item health-related questionnaire was posted at the PWSA (USA) website for access by more than 3000 parents or caregivers in the database registry, and was offered as a voluntary survey (see Questionnaire Form, (Figure 1)). Participation was also solicited from the PWSA (USA) membership through an email link, or, in some cases, as a hard copy sent to families if requested by regular mail. The caregivers of children and adults with PWS (living or deceased) were asked to fill out the survey, even without a history of clots, so as to help learn more about the contributing factors or aspects related to blood clots. The dates of that the survey occurred were between January 2015 to April 2016, with 1067 respondents participating in the study. 

The questionnaire included standard demographic information (e.g., age, gender, weight, height, and PWS genetic subtype) and medical history, such as obesity status, presence or absence of metabolic syndrome, diabetes, edema, kidney problems, hypertension, vasculitis, and growth hormone treatment. Information on blood clots included the onset, location, duration, and severity, with or without a positive family history, vasculitis, or known blood clotting disorders such as Factor V Leiden, prothrombin (Factor II), methylene tetrahydrofolate reductase (MTHFR), Protein S, or Protein C deficiencies, and their treatment. Information about the PWS genetic subtypes and methylation status was requested, as well as PWS deletion subtypes. 

Respondents were probed regarding experiences over the lifetime of their charge, with current biometrics of age, height, and weight; or the last data available if the individual was deceased. Individual responses to the questionnaire were initially screened for errors by the PWSA (USA) staff, who resolved inconsistencies and confirmed answers with respondents prior to analysis. If a history 

of blood clots or bleeding disorders were noted on the questionnaire form, a medical expert (e.g., physician knowledgeable about PWS and specialized in cardiovascular disease (co-author, J.L.)) and/or PWSA (USA; staff member (co-authors, B.M. or C.L.)) made contact with the respondent to clarify any questions and responses prior to data inclusion and analysis. The final de-identified dataset was examined, as well as the descriptive statistics, frequencies, and means generated using SAS statistical software version 9.2 (SAS Institute Inc. Cary, North Carolina, USA). Primary statistical analyses included an analysis of variance (ANOVA) for the continuous data and the chi-squared test for categorical measures.

## 3. Results

A total of 1067 respondents completed the survey describing outcomes for *n* = 554 females and *n* = 513 males with PWS (Table 1). The described PWS subjects had a current mean age of 21.0 ± 14 years with a range of 0 to 63 years, with 502 (47%) being less than 18 years of age. Eight hundred and seventy-five of the 1067 (82%) respondents reported a PWS genetic subtype, with 527 (60%) reporting the typical 15q11-q13 deletion, 325 (37%) reporting maternal disomy 15, and 23 (3%) reporting an imprinting defect similar to the reported frequencies of the genetic subtypes recently described by Butler et al. [6] in a PWS cohort (*n* = 510 subjects) using advanced genetic testing (e.g., high-resolution chromosomal microarrays) in the largest analysis of PWS genetic subtype frequencies to date.

A thrombosis was reported for 38 (3%) PWS cases surveyed, involving 23 females (61%; 4% of females) and 15 males (39%; 3% of males). Of these cases, 33 (87%) events occurred in adults greater than 17 years of age (approximately 6% of adult respondents). The frequency of the blood clots did not differ by gender or PWS genetic subtype, and their occurrence was associated with a significantly greater age, weight, and body mass index (BMI; Table 2). The type of event was identified in 32 cases as a DVT (*n* = 8; 21%), thrombosis (*n* = 7; 18%), embolism (*n* = 4; 11%), PE (*n* = 6; 16%), smaller vein clot (*n* = 6; 16%), and one reported ventricular hemorrhage. Fourteen subjects had blood clots in the leg, five in the lung, and two in the brain, and the source of the blood clot was unknown in 17 subjects. The blood clot resolved in 23 individuals (64%) and recurred in seven (19%) subjects. The blood clot was fatal in four individuals, or 11% of the 38 subjects. Twenty-seven (75%) individuals required hospitalization, and eight had a prior injury or blood clot following a prior surgical procedure. Severe pain was reported in 22 (61%) individuals with PWS, and venous stasis, including leg swelling, reported in 23 (77%) individuals, with discoloration reported in 17 (57%). Medical conditions or treatments at the time of the blood clot formation included obesity in 22 (63%) subjects, 8 (23%) who had diabetes, and 5 (14%) who received growth hormone treatment. 

A summary of the significant risk factors for thromboembolism in PWS is provided in Table 2. These predictors included established relationships with obesity, metabolic syndrome, and renal and cardiopulmonary dysfunction. The odds ratio for a reported clot was greatest when co-occurring with vasculitis (odds ratio (OR) = 36.9; 95% confidence interval (CI) 15.5 to 87.6), which is often associated with skin picking in PWS (OR = 1.9; 95% CI 1.0 to 3.7). Vasculitis was followed by edema (OR = 15.5; 95% CI 7.4 to 32.6) and kidney failure (OR = 14.9; 95% CI 5.3 to 41.9), heart failure (OR = 9.9, 95% CI 3.9 to 24.9), metabolic syndrome (OR = 5.6; 95% CI 1.8 to 17.4), obesity (OR = 5.4; 95% CI 2.1 to 14.1), and atrial fibrillation (OR = 5.4; 95% CI 1.8 to 16.6). Other predictors included behavioral factors (skin picking and smoking) and prior drug treatment with aspirin and blood thinners with a lower frequency. Of interest, previous treatment with growth hormone was associated with significantly reduced risk of thrombosis (OR = 0.2; 95% CI 0.1 to 0.39).

A total of 502 respondents were in the pediatric age group, with a collective mean age of 9.3 ± 5 years and a range of 0–17 years. Of these reports, thrombosis occurred in five (1%) children. The average age for the children with blood clots was 5.6 years, with a range of 0 to 14 years. Three of these children had the 15q11-q13 deletion, and three had a prior history of growth hormone treatment. All five children experiencing thrombosis possessed precipitating risk factors. One child had obesity with metabolic syndrome; two children had a history of skin picking, and one child had edema. One child had a genetic risk factor as a result of a positive family history of blood clots, and developed heart failure with thrombocytopenia.

## 4. Discussion

Blood clots, venous thromboembolism, and/or pulmonary embolism have recently emerged as major medical complications and as contributors to morbidity and mortality in PWS. A health-related questionnaire focused around blood clots was developed by medical experts and researchers in the study of PWS, and administered as a national survey to advance the understanding of and to characterize the frequency of blood clots and related risk factors in this rare obesity-related genetic disorder. Information on the presence or absence of growth hormone treatment, medication use, co-morbid health conditions, and relevant lifestyle factors were also collected. Each event was individually verified and characterized according to age and severity, and whether severe episodes were fatal. 

A summary of the conclusions reached from the above data and from the information provided by each respondent showed that the occurrence of blood clots was predominantly seen in adults at rates of 3% to 4%, higher than the <1% rate reported for the general United States population by the U.S. Center for Disease Control (Beckman et al. [10]). Clot formation in the non-PWS population is known to follow an established male prevalence, which is attributed to gender differences in the hormone levels influential in clot formation. Our failure to identify similar gender differences in PWS cases may be related to disorder-specific neuroendocrine disruption, with associated hypogonadism and sexual immaturity that is characteristic of PWS. The frequency of PWS genetic subtypes (15q11-q13 deletion, maternal disomy 15, and imprinting center defects) provided in the survey was similar to the frequencies of the molecular classes reported by Butler et al. [6], supporting the validity of this voluntarily blood clot-related questionnaire via on-line access or by regular mail over a 15-month period. In addition, our study reports the largest number of individuals with PWS (N = 875) and known genetic subtypes, confirming the established frequencies of PWS molecular classes. PWS genetic subtypes did not appear to influence the probability of a reported thrombosis. 

The risk factors identified in our analyses included excessive weight, metabolic syndrome and cardiovascular illness, as seen in the general population. The relative strength of association with vasculitis, edema, and kidney and heart failure support a progressive disease profile, potentially arising as secondary complications of exogenous obesity complicated by other neuroendocrine disruptions seen in PWS. Vasculitis is a critical risk factor interrelated with both the physiologic and behavioral features associated with PWS, which may be amenable to intervention and strict oversight to reduce skin picking, proactively address skin lesions, and mitigate infections, which may substantially reduce risks. Blood clots in children with PWS were not related to obesity, and appeared to reflect the medical and surgical complications or genetic risk factors related to the family history. 

Growth hormone treatment was associated with a reduced risk of thrombosis in PWS, and may be due to a lower frequency of obese-related or comorbid illnesses, known to contribute to blood clotting events in the general population, and impacted positively by the treatment. The precise contributions of growth hormone treatment to the risk of thrombosis cannot be definitively established from these data, as growth hormone treatment is also a marker for more proactive medical management and vigilant behavioral monitoring in order to prevent the development of obesity not probed in our survey. Furthermore, the access and widespread availability of growth hormone treatment for PWS did not begin until approximately the year 2000, leading to significant differences between adult and child cohorts, impacting on the overall health and wellness in PWS. These cohort-related disparities in medical management, monitoring, and oversite necessitate confirmatory follow-up studies. This dataset and the survey results provide a baseline to monitor future data related to co-morbidities, natural history, survival trends, and causes of death in PWS.

## Figures and Tables

**Table 1:** Sample characteristics for *n* = 1067 total on Prader–Willi syndrome (PWS).

Variables	Overall (*n* = 1067)	Clots (*n* = 38)	No Clots (*n* = 1013)	χ^2^; F	*p*–Value
Female sex	554 (52%)	23 (62%)	531 (52%)		
Male sex	513 (48%)	15 (41%)	498 (49%)	1.3	0.26
**Reported PWS Subtype**	**875 (82%)**	**20 (54%)**	**855 (84%)**		
Deletion Subtype	527 (60%)	12 (60%)	515 (60%)		
UPD15 Subtype	325 (37%)	8 (40%)	317 (37%)		
ID Subtype	23 (3%)	0	23 (3%)	0.58	0.75
Age	21.0 ± 14 years Range: 0–63	32.8 ± 15 years Range: 0–59	20.4 ± 13 years Range: 0–61	31.92	**<0.0001**
BMI	28.9 ± 12 Range: 3.6–104	41.3 ± 18 Range: 13–80	28.2 ± 12 Range: 4–104	42.27	**<0.0001**
Height	57.1 ± 10 inches Range: 17–77	58.8 ± 10 inches Range: 21–68	57.0 ± 10 inches Range: 17–77	0.96	0.33
Weight	144 ± 78 lb Range: 5–500	217 ± 104 lb Range: 8–490	140 ± 75 lb Range: 5–492	35.75	**<0.0001**

The sample characteristics are presented for the total sample (overall), those with a reported thromboembolism (clots), and without a reported thromboembolism (no clots). Thromboembolisms included all of the reported deep or small vein thrombosis events, pulmonary embolism, or other reported embolism. Chi-square test or analysis of variance (ANOVA) were used to test for differences between individuals with and without blood clots by gender, PWS subtype, age, weight, height, and body mass (BMI). A total of *n* = 502 (47%) were <18 years of age.

**Table 2:** Frequency and analysis of risk factors for thromboembolism in Prader–Willi syndrome.

Variable	Clots, *N* (%)	No Clots, *N* (%)	χ^2^	*p*–Value
**Medical Findings and History**				
Obesity *	32 (86%)	539 (54%)	15.2	**<0.0001**
Edema *	28 (74%)	151 (15%)	86.6	**<0.0001**
Skin Picking *	19 (51%)	354 (35%)	4.0	**0.045**
Vasculitis *	13 (48%)	22 (2.5%)	150.0	**<0.0001**
Metabolic syndrome	5 (36%)	56 (9%)	11.3	**<0.0008**
Hypertension	12 (32%)	121 (12%)	12.9	**<0.0003**
Diabetes	7 (20%)	117 (12%)	1.92	0.16
Heart Failure	7 (19%)	23 (2.3%)	35.1	**<0.0001**
Kidney function	6 (17%)	14 (1.3%)	44.6	**<0.0001**
Smoker *	5 (13%)	36 (4%)	9.0	**<0.0028**
Atrial fibrillation *	4 (12%)	23 (2.4%)	10.9	**<0.001**
Cancer	4 (10.5%)	8 (0.79%)	30.6	**<0.0005 (Exact)**
Atherosclerosis	2 (6.3%)	2 (0.21%)	28.1	**<0.0055 (Exact)**
Antiphospholipid antibody syndrome *	1 (4.5%)	2 (0.22%)	12.8	0.004 (Exact)
Thrombocythemia *	1 (4.3%)	7 (0.8%)	3.3	0.18 (Exact)
Bone marrow disorder *	1 (3.1%)	5 (0.53%)	3.4	0.17 (Exact)
**Family History**				
Clotting *	9 (24.3%)	137 (14.4%)	2.8	0.09
Factor V deficiency *	2 (8%)	16 (2%)	4.7	**0.03**
MTHFR deficiency *	1 (5%)	10 (1%)	1.9	0.16
Factor II deficiency *	0	4		NA
Protein S deficiency *	0	5		NA
Protein C deficiency *	0	5		NA
**Prior Treatments**				
Growth hormone (ever)	14 (37%)	751 (75%)	26.4	**<0.0001**
Blood Thinners	25 (67%)	19 (2%)	370	**<0.0001**
Hypothyroidism	8 (24%)	183 (18%)	0.53	0.46
Aspirin	8 (22%)	30 (3%)	36.6	**<0.0001**
Hyperthyroidism	2 (6%)	6 (0.6%)	11.3	0.028 (Exact)
Adrenal Insufficiency	1 (3%)	40 (4%)	0.09	0.75

Summary of the characteristics and differences between the subjects with a reported thromboembolism (clots) and without a reported thromboembolism (no clots). Thromboembolisms included all of the reported deep or small vein thrombosis events, pulmonary emboli, or other reported emboli. Sample *n* = 1067; *n* = 38 individuals with blood clots: reported frequency of variable by clotting history and statistical analyses using a chi-squared test. * Represent factors that could contribute to the development of blood clots in an individual.
